# Partially Disordered Structure in Intravirus Coat Protein of *Potyvirus Potato Virus A*

**DOI:** 10.1371/journal.pone.0067830

**Published:** 2013-07-03

**Authors:** Alexander L. Ksenofontov, Viiu Paalme, Alexander M. Arutyunyan, Pavel I. Semenyuk, Natalia V. Fedorova, Reet Rumvolt, Ludmila A. Baratova, Lilian Järvekülg, Eugeny N. Dobrov

**Affiliations:** 1 Belozersky Institute of Physico-Chemical Biology, Lomonosov Moscow State University, Moscow, Russia; 2 Institute of Gene Technology, Tallinn University of Technology, Tallinn, Estonia; 3 Competence Center for Cancer Research, Tallinn, Estonia; Russian Academy of Sciences, Institute for Biological Instrumentation, Russian Federation

## Abstract

*Potyviruses* represent the most biologically successful group of plant viruses, but to our knowledge, this work is the first detailed study of physicochemical characteristics of potyvirus virions. We measured the UV absorption, far and near UV circular dichroism spectra, intrinsic fluorescence spectra, and differential scanning calorimetry (DSC) melting curves of intact particles of a *potato virus A* (PVA). PVA virions proved to have a peculiar combination of physicochemical properties. The intravirus coat protein (CP) subunits were shown to contain an unusually high fraction of disordered structures, whereas PVA virions had an almost normal thermal stability. Upon heating from 20°C to 55°C, the fraction of disordered structures in the intravirus CP further increased, while PVA virions remained intact at up to 55°C, after which their disruption (and DSC melting) started. We suggest that the structure of PVA virions below 55°C is stabilized by interactions between the remaining structured segments of intravirus CP. It is not improbable that the biological efficiency of PVA relies on the disordered structure of intravirus CP.

## Introduction

*Potyviruses* are the largest and most economically important group of plant viruses [1]. As far as we know, this work is the first attempt of a more or less detailed analysis of physicochemical characteristics of any *potyvirus*. This could be due to the difficulties in obtaining sufficient amounts of the pure material. On the other hand, the new generation of instruments for physicochemical measurements can work with much lower sample concentrations.

In spite of almost sixty years of studies, high-resolution structural information for helical plant viruses (HPV) is still unavailable. Even for *tobamoviruses*, the X-ray fiber diffraction [2] and cryoelectron microscopy [3] data with a resolution of 3–5 Å are insufficient for elucidating the mechanisms of virus assembly *in vitro* and *in vivo*, especially since important differences between the results obtained by these two methods exist. For *potyviruses,* the best achievement in this field to date is 14 Å resolution fiber diffraction and cryoelectron microscopy data from Dr. Stubbs’s laboratory [4]. These data made it possible to estimate only overall dimensions of coat protein (CP) subunits in virions. The model of *potato virus A* (PVA) CP structure presented in our 2001 publication [5] remains the only more or less detailed model of *potyvirus* structure. In this context, other indirect methods may contribute to solving these problems.

This communication presents the results of our study of the properties of PVA virions and isolated CP with the help of UV absorption, far UV and near UV circular dichroism (CD) spectroscopy, fluorescence spectroscopy, and differential scanning calorimetry (DSC).

## Materials and Methods

### Virus Purification

A method elaborated by us previously [5] was used to purify PVA isolate B11 (originally isolated in Germany) with minor modifications. Below is a brief survey of the most important phases of PVA purifying. The virus was purified from systemically infected leaves of *Nicotiana benthamiana* harvested 18–21 days after inoculation. The leaves were pulverized in a Waring blendor with 0.05 M phosphate buffer, pH 8.0, containing 0.01 M DIECA, 0.005 M EDTA, and 1% sodium sulfite (2 ml of buffer per g of leaf tissue).

After a low speed centrifugation (LSC) (8,000 g, 5°C, 20 min), the supernatant was stirred with Triton X-100 (1%, vol/vol) for 1 h at 4°C. Then, 50 g/l polyethylene glycol 6000 and 12 g/l NaCl were added and the mixture was stirred at 4°C for 1.5 h. The precipitate was recovered by LSC and resuspended in 0.05 M borate buffer, pH 8.0. Insoluble material was removed by another LSC and the supernatant was subjected to high-speed centrifugation through 20% (vol/vol) sucrose cushion (150,000 g, 5°C, 2.5 h). The pellet was resuspended in 0.05 M borate buffer, pH 8.0, and subjected to another differential centrifugation. The final pellet was resuspended in 0.05 M borate buffer pH 8.0.

PVA CP was isolated using the standard LiCl method [6].

The purity of the preparations was checked by sodium dodecyl sulfate-polyacrylamide gel electrophoresis (SDS-PAGE) [7], electron microscopy, and UV absorption spectra.

### Electron Microscopy

Carbon coated grids (S160-3, Agar Scientific) were placed onto PBS-diluted drops, incubated for 1 h at room temperature, washed twice with H_2_O, contrasted with 2% uranyl acetate for 5 min at room temperature, and dried. The samples of infected leaves sap were examined under a Zeiss 10A transmission electron microscope with a magnification of 20,000.

### UV Spectra Measurements

The absorption spectra in the 240–338 nm range were measured in cells with an optical path of 1 cm using a Hitachi UV-2600 spectrophotometer. True absorption (E) spectra of light-scattering suspensions were calculated by the extrapolation method [8] using different computer programs [9], [10]. The spectral region of 320−338 nm was used for extrapolation. Virus concentration was estimated spectrophotometrically assuming an E^0.1%^_260 nm_ of 2.3 optical units.

### CD Spectroscopy

CD spectra in the 190−250 nm (far UV) and 260−340 nm (near UV) regions were recorded on Chiroscan CD spectrometer (Applied Photophysics, United Kingdom) in 1-mm and 1-cm cells, respectively. Virus concentrations of 0.05−0.15 and 0.5−1 mg/ml in 5 mM phosphate buffer, pH 7.0, were used for far and near UV CD measurements, respectively [11]. Far UV spectra were calculated in ellipticity ([Θ]) per mole of amino acids; near UV CD spectra, in Δε values. The RNA content of virions was taken to be 6% [12]; the mean amino acid molecular weight, 110 Da; and mean ribonucleotide molecular weight, 320. The spectra were recorded at 0.5–1.0 nm/s. The measured spectra were smoothed with the instrument software. Each point was measured for 1 s.

### Fluorescence Measurements

Fluorescence spectra of protein solutions were measured in a 0.1-cm quartz cell at various temperatures with a FluoroMax-3 spectrofluorometer (Horiba Jobin Yvon, USA). Fluorescence of 0.02−0.05 mg/ml virions in 5 mM phosphate buffer, pH 7.0, was excited at 280 nm. The samples were loaded in the cell and heated to 75°C at the rate of 1°C/min. The emission spectra were recorded from 300 to 400 nm.

### Differential Scanning Calorimetry

DSC experiments were performed on a DASM-4 microcalorimeter (Biopribor, Russia) with 0.47-mL platinum capillary cells at a scanning rate of 1°C/min. The second heating was used as the instrument baseline because of the irreversible denaturation observed for the samples investigated. The chemical baseline was calculated and subtracted using the Origin 1.16 software.

### Secondary Structure Prediction

We have chosen the five best-known programs for secondary structure prediction according to the critical assessment of protein structure prediction (CASP) at http://predictioncenter.org. These programs included GOR4 (http://npsa-pbil.ibcp.fr/cgi-bin/secpred_gor4.pl), APSSP2 (http://www.imtech.res.in/raghava/apssp2/), SCRATCH (SSpro v. 4.5) (http://scratch.proteomics.ics.uci.edu), JPred (Jnet) (http://www.compbio.dundee.ac.uk/www-jpred/), and PSIpred (http://bioinf.cs.ucl.ac.uk/psipred/).

The presence of intrinsically disordered (ID) segments in the isolated PVA CP was analyzed with the help of the following four available predictors based on different algorithms and prerequisites: POODLE-S (http://mbs.cbrc.jp/poodle/poodle.html), Iupred (http://iupred.enzim.hu/), VL3H (http://www.dabi.temple.edu/disprot/predictor.php), and FoldUnfold (http://bioinfo.protres.ru/ogu/).

## Results

**Figure 1:**
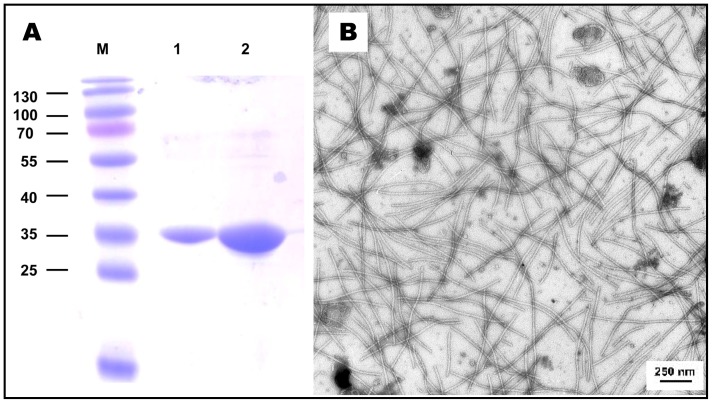
Characteristics of purified PVA B11 virions. (**A**) PAGE; preparations were separated by discontinuous Tris-glycine 13% SDS-PAGE. For Mw determination Page Ruler Prestained Protein ladder (Fermentas SM0671) was used for Mw determination (lane M). Purified virus aliquots of 1 µg (1) and 10 µg (2) per lane were used. (**B**) Electron microscopy of sap from PVA-infected *N. benthamiana*; magnification ×20,000.

**Figure 2:**
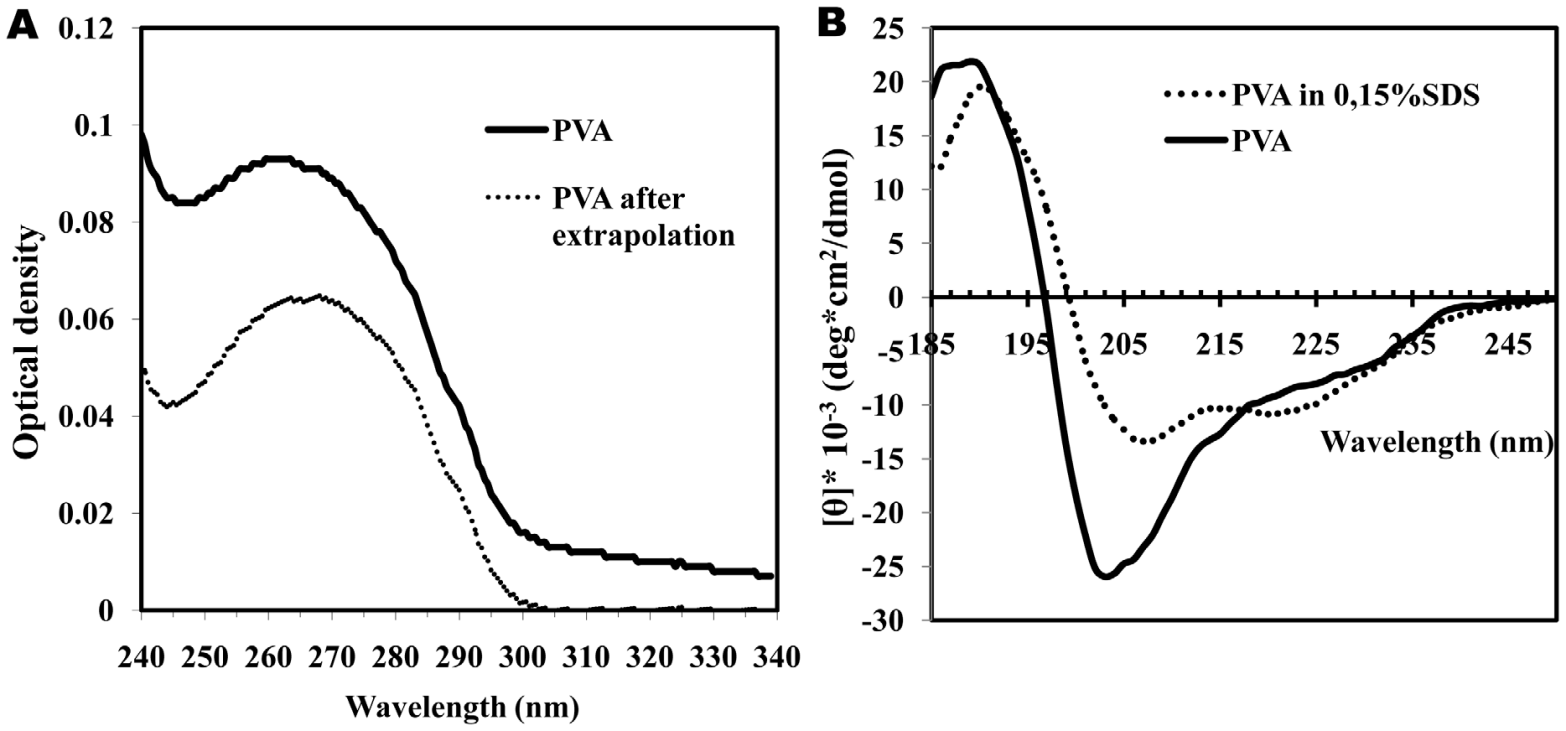
UV absorption (A) and far UV CD (B) spectra of PVA virions. (**A**) Directly measured UV absorption spectrum of intact PVA virions in 10 mM phosphate buffer, pH 7.0 (solid line) and scattering-corrected (dotted line) spectrum are shown. (**B**) Far UV CD spectra of intact (solid line) and 0.15% SDS-disrupted (dotted line) PVA virions in 10 mM phosphate buffer were measured in 1-mm cells at 25°C at PVA concentration of 0.14 mg/ml.

PVA-B11 preparations were grown in *N. benthamiana* plants and purified as described in Materials and Methods. An electron micrograph of the infected leave sap is shown in Fig. 1B; a UV absorption spectrum, in Fig. 2A; and the results of SDS-PAGE analyses of the intact virus sample, in Fig. 1A. As observed many times previously, PVA CP had anomalously low electrophoretic mobility corresponding to about 35 kDa, while the real Mw of PVA CP is 30.26 kDa [5]. In addition, no degraded CP is visible in our PVA preparations.

A significant difference between *potexviruses* and PVA is that many *potexviruses* can be obtained in much larger amounts than PVA. The available quantities of PVA (and especially of its CP) were sufficient only for UV absorption, CD and fluorescence spectra, and DSC melting experiments.

### UV and CD Spectra

Some of our PVA preparations had too high E_260_/E_280_ ratio (1.35–1.4) instead of the predicted ∼1.25, and this ratio did not decrease after repeated high-speed centrifugations. We suggest that PVA virions can adsorb RNA molecules on their surface, which leads to an overestimation of the sample concentration. In all experiments described below, only the PVA preparations with the normal E_260_/E_280_ ratio (∼1.25) were used (Fig. 2A). Besides, our PVA preparations had unusually high stability and did not lose their characteristics after storage at +4°C in neutral 10 mM phosphate or Tris buffers for several weeks without any additives (chloroform, azide, etc.).

First of all, we measured the far UV CD spectrum of intact PVA virions (Fig. 2B).The intact virus spectrum had a high [θ]_-max_ value, and the main negative maximum was detected at 203 nm instead of the usual 208 nm, which suggests an unusually high content of disordered structures. As far as we know, PVA is the only HPV with such a shift of the main negative maximum in the far UV CD spectrum. Some (but not all) helical plant viruses possess anomalous far UV CD spectra, and the nature of this anomaly was discussed many years (for instance [14], [16]). The abnormal character of these spectra makes it impossible to use any programs predicting protein secondary structure. This also applies to the far UV CD spectrum of intact PVA. After the disruption with 0.15% SDS at room temperature, this maximum shifted to normal (208 nm) position and its intensity ([θ]_-max_) dropped from –26000° to –15000°. This may mean that PVA CP acquires a more ordered structure after the disruption with 0.15% SDS. The intact PVA spectrum could not be processed by the web service K2D2, which estimates protein secondary structure from CD data [13], but after the virion disruption with SDS this program gave the values of 34%, 18%, and 48% for α-helical, β-sheet, and unordered structures, respectively.

**Figure 3:**
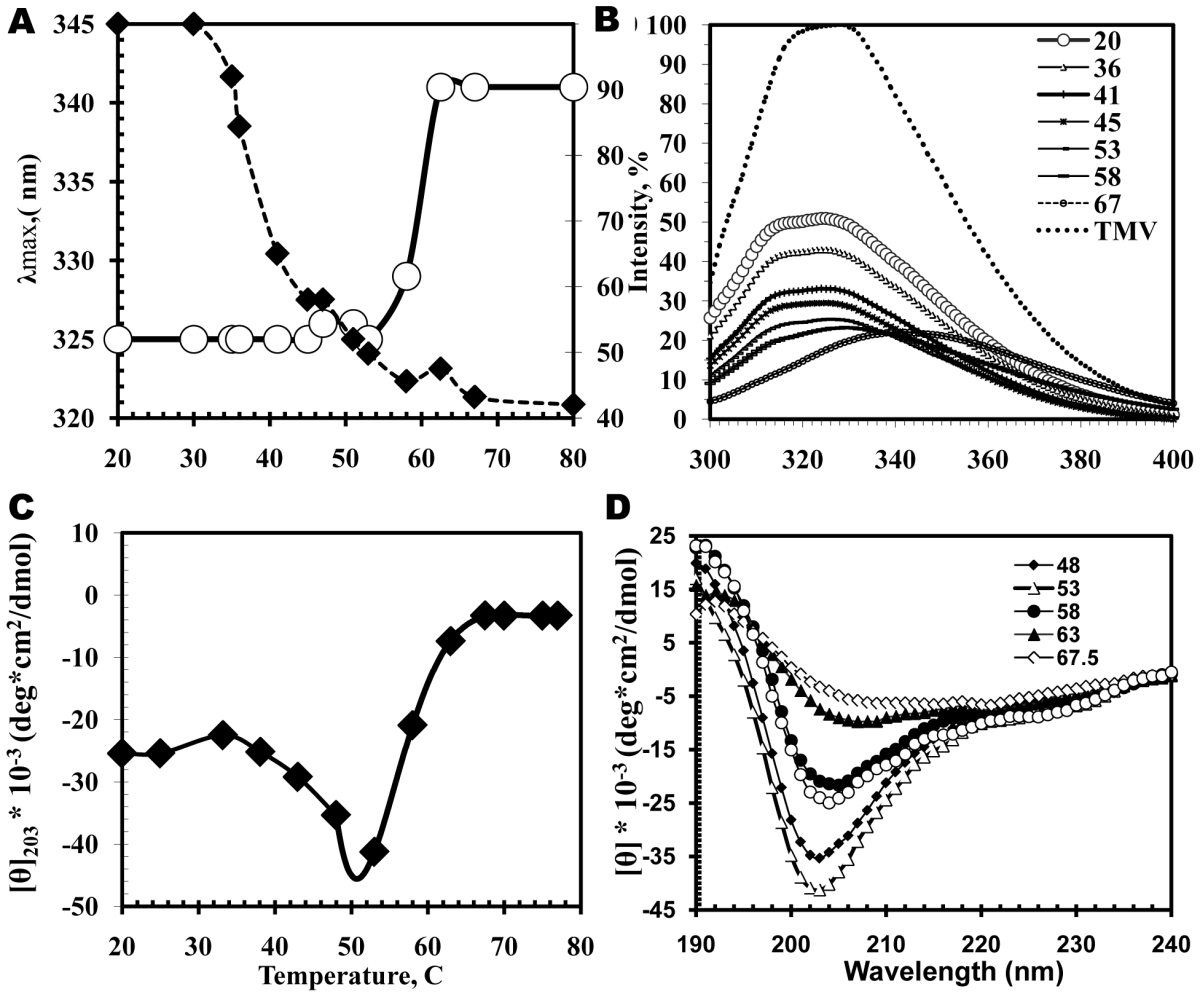
Thermal denaturation of intravirus PVA CP controlled by fluorescence (A and B) and far UV CD (C and D). Concentration and buffer are shown in Fig.2. (**A**) Temperature dependences of fluorescence maximum position (circles) and intensity (diamonds); (**C**), Temperature dependences of [θ]_203._ (**B** and **D**) Complete spectra at indicated temperatures.

Next, we used far UV CD data to analyze the process of thermal denaturation of intravirus PVA CP (Fig. 3C, D). (The results of DSC experiments demonstrate that PVA virion melting occurs at 55°C). Upon heating from room temperature to 55°C, the intensity of the negative maximum increased sharply: from –26000° to –45000°. This means that heating in this temperature range further increased the unordered structure content. The [θ]_203_ value reached its maximum before the virion disruption at 55°C (see Fig. 3). Upon further heating, disrupted virions aggregated and precipitated.

**Figure 4:**
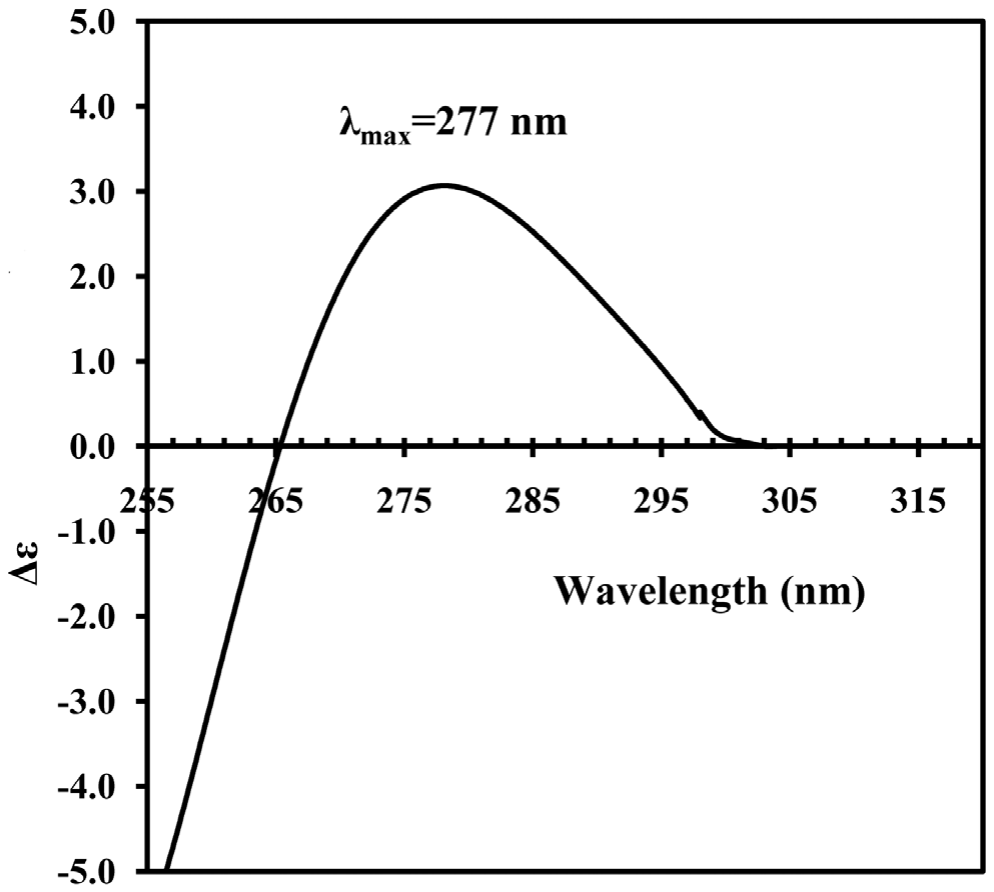
Near UV CD spectrum of intact PVA virions in 10 mM phosphate buffer, pH 7.0. Spectrum was measured at 25°C in 0.5-cm cells at PVA concentration of 0.6 mg/ml.

We also measured the near UV (250–320 nm) CD spectrum of PVA virions. Despite low RNA content of helical plant viruses (5–7%), intravirus RNA contributes most to these spectra [11]. The shape and position of the maximum in the near UV CD spectrum of PVA virions (Fig. 4) were similar to those of other helical plant viruses [8], but the intensity of this spectrum (Δε∼3 units) was exceptionally low. Such intensity (and the position of the maximum) are similar to those of thermally denatured isolated single-stranded RNA [14], [15]. On the basis of the data for *tobamoviruses*, we suggested [11] that the RNA contribution to their near UV CD spectra constitutes about 60–70%. Limited amounts of the material did not allow us to measure the near UV CD spectrum of isolated PVA CP. In 2008, we demonstrated that isolated CP of *potato virus X* (PVX) had negative (although not too strong) near UV CD spectrum [16]. In any case, the near UV CD spectrum of intravirus PVA RNA should be more or less close to the spectrum of denaturated isolated single-stranded RNAs and may also indicate some kind of structural disorder. Therefore, this characteristic should be added to the list of PVA structural peculiarities.

### Fluorescence Spectra

We studied the tertiary structure of intravirus PVA CP by measuring their intrinsic fluorescence spectra in the 300 to 400 nm region (Fig. 3A,B). PVA (and its CP) are not very convenient for this kind of studies, because PVA CP contains a significant fraction of aromatic amino acids largely represented by Tyr in ratio to Trp of three to one (9Y and 3 W). Accordingly, the intrinsic fluorescence spectrum of PVA virion had two maxima at 314 and ∼327 nm. The maximal intensity in the PVA spectrum was more than two times lower than that of TMV virions, which serve as the standard of proteins with high intrinsic fluorescence (Fig. 3B). Upon stepwise heating, the fluorescence intensity of PVA virions dropped to about 50% of the initial value (Fig. 3A,B). The ratio between the maxima at 314 and 327 nm remained more or less constant upon heating to about 55°C. This may mean that the environment of Trp and Tyr residues changes in parallel. These data suggest that the fluorescence characteristics of PVA virion correspond to a not too stable tertiary structure in the intravirus CP, and this structure does not change too much after heating up to 55°C. At higher temperatures, PVA virion starts to disrupt (see Figs 3 and DSC experiments below), and the observed effects resulted from virion precipitation.

**Figure 5:**
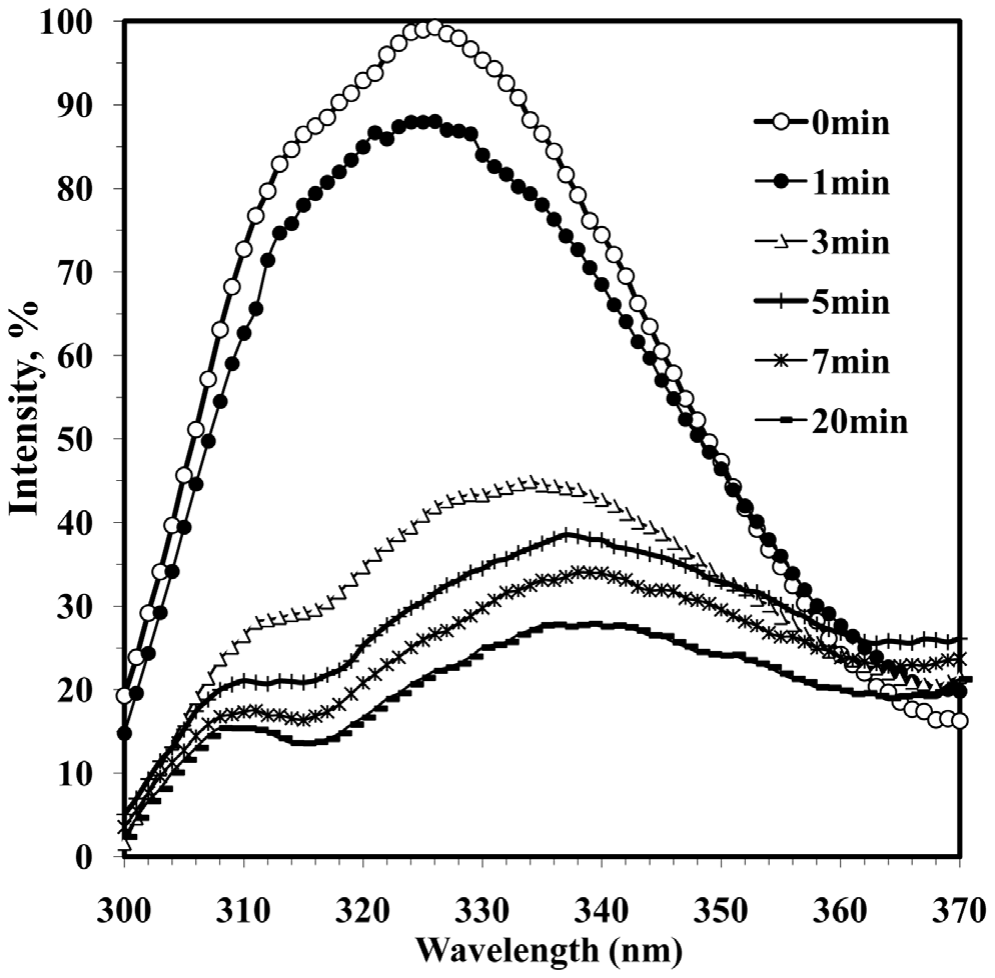
Intrinsic fluorescence spectra of intact and 0.15% SDS-disrupted PVA virions. Excitation by 280 nm light at 25°C. Duration of incubation in 0.15% SDS are shown in the upper right corner. Sample concentration was 0.03 mg/ml.

In the presence of 0.15% SDS, the changes in PVA fluorescence had a different pattern (Fig. 5). After 10 min incubation with 0.15% SDS at 25°C (see also CD in Fig. 2), the virions were disrupted and the fluorescence intensity dropped about four times, the main maximum shifted from 326 to 338 nm, and an additional maximum appeared at 312 nm, thus, testifying to a much more complete loss of tertiary structure then after heating. This suggests that the intravirus CP tertiary structure was largely maintained by intersubunit interactions. These interactions can be attributed to the secondary structures of intravirus PVA CP that survived heating up to 55°C.

### Differential Scanning Calorimetry

**Figure 6:**
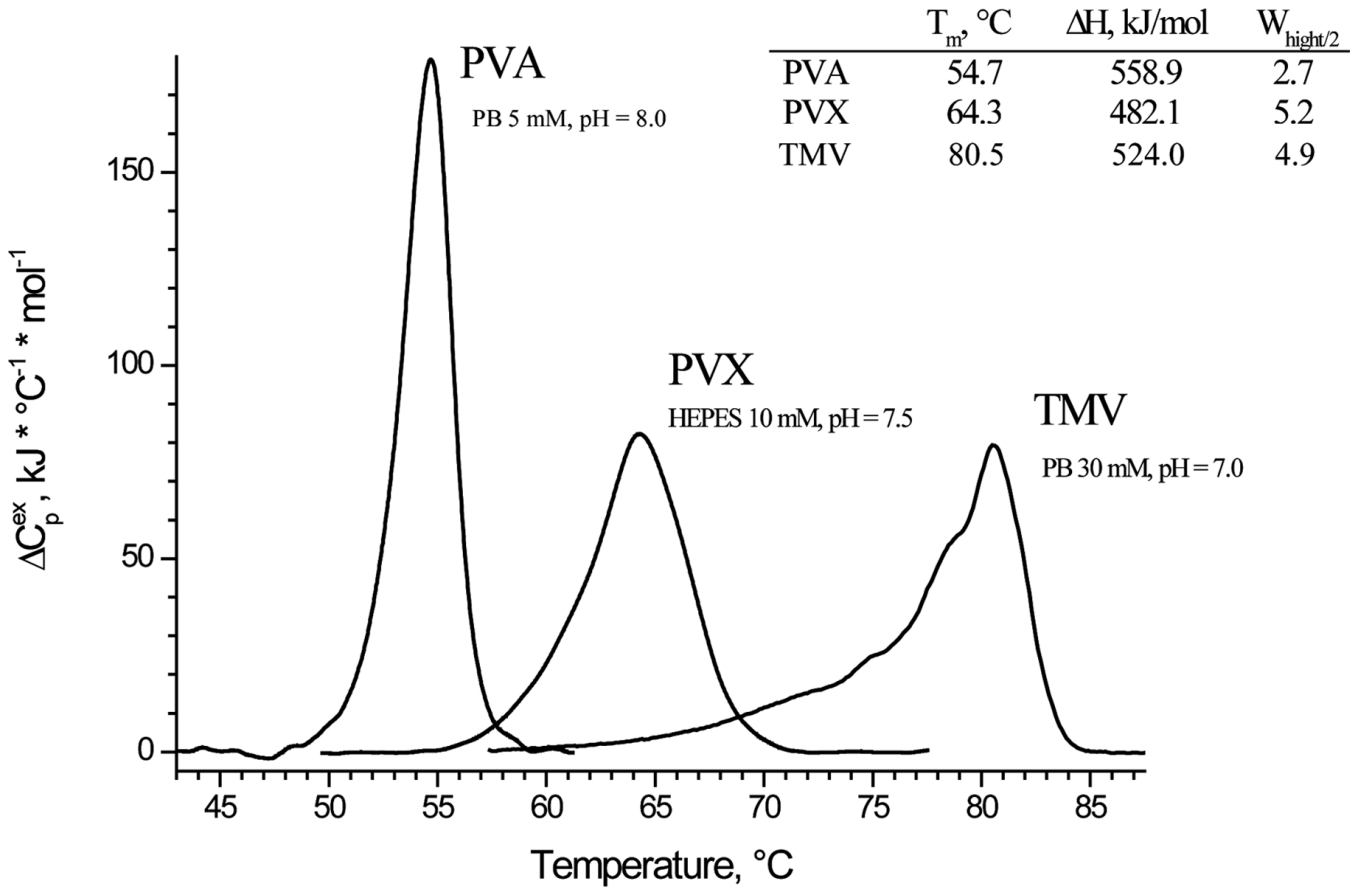
DSC melting curve for intact PVA virions in comparison with those of rod-shaped TMV virions and filamentous PVX virions in 10 mM phosphate buffer, pH 7.0. Melting temperatures (T_m_,°C), enthalpy values (ΔH), and width at half height (W_hight/2_ ) are shown in the upper right corner.

We also measured DSC melting curves for the intact PVA virions (Fig. 6). To our knowledge, it is the first published DSC melting profile for any of *potyviruses*. Such curves were reported for rod-like TMV and flexuous PVX [16], [17], and we also present them in Fig. 6. The main advantage (or disadvantage) of DASM calorimeters is their low sensitivity to protein/virus aggregation in the course of heating.

The curves were obtained under similar conditions (concentration, heating rate, and buffer nature) for all three viruses, and therefore can be directly compared. The T_m_ values proved to be more or less similar in the DSC melting curves for all three viruses (Fig. 6). TMV had the highest temperature stability (T_m_ = 80.5°C), while PVA was the least stable (T_m_ = 55°C). In contrast to other HPV, the DSC melting curve for intact PVA had the width typical for proteins (W_hight/2_ = 2.7°), while those for intact TMV and potato virus X featured abnormally large widths (W_hight/2_ = 5–7°), which can be due to some kind of virion heterogeneity [17].

In spite of limited amounts of the material, we tried to measure DSC curves for isolated PVA CP and observed no reliable heat absorption peaks in the 5 to 95°C interval. Regrettably, we cannot be certain that it is not a measurement artifact. On the other hand, the T_m_ for isolated TMV CP is as low as 40°C [17], and that for isolated PVX CP is 35°C [16]. Hence, free PVA CP can indeed lack any meltable structures, and thus, support our suggestion that isolated PVA CP in 10 mM phosphate buffer has a significant fraction of disordered structures.

## Discussion

The results of the present work allow us to suggest that the structure of PVA virions (and possibly of other potyviruses) is characterized by a peculiar structure of its intravirus CP, which contains a significant fraction of disordered segments. The proportion of such segments cannot be quantified from the available CD and fluorescence spectroscopy data. It increases upon virion heating (before the particles start to disrupt at ∼55°C, Figs. 3 and 6) and drops after PVA virion disruption at room temperature in 0.15% SDS. K2D2 program [13] gives values of 34% for α-helices, 18% for ß-strands, and 48% for disordered structures for free PVA CP in 0.15% SDS. After heating from 20°C to ∼55°C, the disordered fraction in intravirus PVA CP increases considerably through the melting of some α-helical segments (without the virion disruption).

The main question raised by these data is what kind of interactions stabilize PVA virions at room temperature and upon heating to about 55°C? Low [θ]_218_ values in the CD spectra of PVA virions cannot be considered a proof of the absence of ß-type structures in intravirus CP. Low predictive accuracy of CD data for evaluating the content of ß-type structures in proteins is well known [18], especially in the presence of a strong negative maximum in the spectra. The appearance of a significant fraction of (intersubunit?) ß-type structures upon the transition from free to the intravirus CP has been recently reported for rod-like virions of Barley stripe mosaic virus [10]. We have no grounds to believe that intravirus PVA CP can contain a significant fraction of polyproline II helix [19].

We used five popular secondary structure prediction programs to analyze PVA CP (see Table 1). It should be stressed that these methods forecast secondary structure for isolated proteins. All these programs predict high content of random coil structure (from 40 to 66%) in free PVA CP; α-helix content in this protein was evaluated as 25 to 45%. However, all five programs forecast the presence of an appreciable fraction of β-structures (from 10 to 16%) in the protein (in our 2001 paper [5], we predicted 16% of β-structures in intravirus PVA CP). Therefore, despite the absence of a significant [θ]_218_ signal in the CD spectra, we cannot exclude the presence of some fraction of β-structures in intravirus PVA CP as well as its participation in the stabilization of the virion structure. These structures may remain intact after heating to 55°C and contribute to the virion integrity.

**Table 1:** *Potato virus A* coat protein secondary structure prediction.

	Prediction programs	α-helix (%)	β-strands (%)	Random coil (%)
**A**	**GOR4**	45	14	40
**B**	**APSSP2**	36	16	48
**C**	**SCRATCH (SSpro v 4.5)**	34	13	53
**D**	**Jpred(Jnet)**	26	16	57
**F**	**PSIPRED V3.3**	23	10	66
	**Average values**	**33±8**	**14±2**	**53±9**

**Figure 7:**
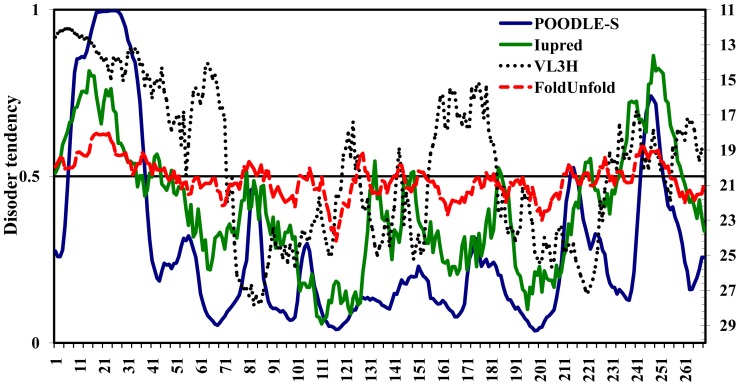
Prediction of folded and unfolded regions in isolated PVA CP was performed using the POODLE-S, Iupred, VL3H, and FoldUnfold programs.

We analyzed the presence of intrinsically disordered (ID) segments in isolated the PVA CP with the help of POODLE-S, Iupred, VL3H, and FoldUnfold programs (Fig. 7). Sequences with the disorder tendency value below 0.5 (or 20.7 for FoldUnfold; red line, right ordinate) were considered as disordered in this figure. All these programs see much disorder in the isolated PVA CP, especially, in the N-terminal (and C- terminal?) regions.

Partially disordered structures in CP subunits in PVA virions can conceivably explain the failure to reach high resolution for *potyviruses* and *potexviruses* in cryoelectron microscopy and X-ray fiber diffraction studies from Dr. Stubbs laboratory [4], [20]. Possible particle heterogeneity in different or even the same PVA preparations can also underlie these problems.

Thus, the results obtained in the present work testify to an unusual structure of intravirus PVA CP. An unusual position of negative maximum ([θ]_203_ ), an increase in disordered structure content upon heating up to 55°C without the virion disruption, a relatively low fluorescence intensity and its relatively small decrease upon heating (without any significant changes in the position of the maxima), a very low value of Δε_max_ in near UV CD spectrum, and an unusually narrow PVA DSC melting curve all suggest the presence of a high proportion of disordered CP structures in the PVA virions. We suppose that these structures are stabilized by intersubunit interactions between α-helixes (and ß-structures?) that survived heating to 55°C. At the present, we can say nothing about the location of these putative “crosslinks” in PVA CP structure, which will be the aim of our future studies.

The concept that intrinsically disordered (ID) protein sequences play the major role in different biochemical processes has become almost universally accepted [21], [22]. According to this concept, the presence of ID regions in proteins (among other things) greatly enlarges the range of their specifically recognized partners. This “promiscuity” may apply not only to the interactions with other proteins but also to ID sequence ability to interact with other cellular components (including different membranous structures). It is now known [23] that, in the course of infection, *potyviruses* interact with many different protein and membranous components of plant cells, and the presence of a significant fraction of ID sequences may be one of factors of their spectacular biological efficiency.

That fact that PVA virions contain, in addition to CP and VPg, two nonstructural proteins, helper component-proteinase and cylindrical inclusion protein, can also indicate that this CP is an ID protein [24], [25].

The unusually high stability of our PVA (strain B11) preparations stored at +4°C observed in this work can also be mediated by disordered structures in the virions and the formation of specific aggregates in solution due to the interactions between surface located disordered regions of intravirus PVA CP.

As far as we know, PVA is the first helical plant virus, whose intravirus CP supposedly contains a significant fraction of ID sequences. If so, PVA virions should resemble internal ribonucleoprotein of paramixoviruses (also minus-RNA containing viruses) [26], [27]. In the case of paramixoviruses, this ribonulceoprotein is within a complex and stable lipoprotein envelope; while in the case of PVA, it is exposed to the environment. In this situation, PVA virions should provide sufficient stability to the intravirus CP to secure the virion survival. Insufficient capabilities of direct methods concerning potyviruses were discussed in the Introduction. Even our last resort, Raman microscopy [28], did not help so far due to high sample fluorescence, although some progress can be reached here.

A couple of months ago, a very important study from Dr. Steinmetz laboratory reported an anticancer activity in preparations of a fairly close relative of PVA, *potexvirus* PVX [29]. A similar activity should be checked in PVA and other *potyviruses.*
